# Therapeutic Drug Monitoring of Antimicrobial Drugs in Neonates: An Opinion Article

**DOI:** 10.1097/FTD.0000000000000919

**Published:** 2021-08-09

**Authors:** Daan J. Touw, John N. van den Anker

**Affiliations:** * Department of Clinical Pharmacy and Pharmacology, University Medical Center Groningen;; † Department of Pharmaceutical Analysis, Groningen Research Institute of Pharmacy, University of Groningen, Groningen, the Netherlands;; ‡ Division of Clinical Pharmacology, Children's National Hospital, Washington, DC;; § Department of Paediatric Pharmacology and Pharmacometrics, University Children's Hospital, University of Basel, Basel, Switzerland; and; ¶ Intensive Care and Department of Pediatric Surgery, Erasmus Medical Center-Sophia Children's Hospital, Rotterdam, the Netherlands.

**Keywords:** TDM, neonate, antibiotic, antifungal, monitoring

## Abstract

Neonatal infections are associated with high morbidity and mortality rates. Optimal treatment of these infections requires knowledge of neonatal pharmacology and integration of neonatal developmental pharmacokinetics (PKs) of antimicrobial drugs in the design of dosing regimens for use with different gestational and postnatal ages. Population PK and pharmacodynamic models are used to personalize the use of these drugs in these fragile patients. The final step to further minimize variability in an individual patient is therapeutic drug monitoring (TDM), where the same population PK/pharmacodynamic models are used in concert with optimally drawn blood samples to further fine-tune therapy. The purpose of this article is to describe the present status and future role of model-based precision dosing and TDM of antimicrobial drugs in neonates.

**Methods:**

PubMed was searched for clinical trials or clinical studies of TDM in neonates.

**Results:**

A total of 447 articles were retrieved, of which 19 were concerned with antimicrobial drugs. Two articles (one aminoglycoside and one vancomycin) addressed the effects of TDM in neonates. We found that, in addition to aminoglycosides and vancomycin, TDM also plays a role in beta-lactam antibiotics and antifungal drugs.

**Conclusions:**

There is a growing awareness that, in addition to aminoglycosides and vancomycin, the use of beta-lactam antibiotics, such as amoxicillin and meropenem, and other classes of antimicrobial drugs, such as antifungal drugs, may benefit from TDM. However, the added value must be shown. New analytical techniques and software development may greatly support these novel developments.

## INTRODUCTION

A standard dose and dosing interval are chosen for most drugs when drug therapy is prescribed to a patient. For example, in a patient with hypertension, a diuretic is initiated at a fixed dose, and after a few weeks, blood pressure is evaluated, and the dose is adjusted based on the obtained effect and side effects.

Dose and effects are investigated during various phases in drug development, and the results of clinical trials and dosing strategies are documented in the registration files. In addition, a synopsis is published in the summary of product characteristics (SmPCs) and product leaflet, which medical professionals use as references in their choice of drug and dose. For many drug classes, this strategy is based on clinical investigations with large groups of patients and usually represents the average effect in the average patient. This average patient can be characterized by the average clearance and volume of distribution of the drug. However, not all patients are average, and not all drugs are harmless if the dose is chosen by the rule of thumb, especially when the clinical effect of the drug cannot be easily monitored, as in the blood pressure–lowering drug example. For these drugs, a personalized dosing method may be more appropriate, and measuring the blood concentration of the drug is one method with which to adjust the dose based on a predetermined target concentration for the drug and a pharmacokinetic (PK)/pharmacodynamic (PD) model to initiate therapy. This method of choosing the correct dose for the initial use by an individual patient could be called “goal-oriented, model-informed, precision dosing,” and it is usually followed by a dose adjustment using predefined target concentrations, which is known as therapeutic drug monitoring (TDM).

Newborn infants are especially vulnerable, and finding the optimal dose to treat these fragile humans is extremely challenging. Neonates are not small adults or children, and the same is true for their PKs. In humans, organs and their functions, such as hepatic metabolic capacity and renal clearance, undergo a steep but variable maturation process after birth. Most hepatic phase I and phase II enzymes, which are responsible for drug metabolism, reach maturity after 1 year of age; unfortunately, there is no easy marker to evaluate the developmental stage of the liver.

Neonatal renal function increases after birth. At birth, the glomerular filtration rate is low (10–20 mL/min/1.73 m^2^) and increases to 30 mL/min/1.73 m^2^ at the age of 2 weeks.^1^ Preterm neonates have fewer glomeruli than full-term neonates. This is reflected in the half-life of any renally cleared drug. When designing drug dosage schemes for neonates, developmental considerations must be taken into account. Common descriptors used for maturation include gestational age (GA) and postnatal age. Other descriptors include body weight (BW) and body surface area. However, not every neonate has the same speed of organ development, and illness or drug treatment may affect organ function in addition to the already existing intraindividual variability. In this context, establishing the correct renal function in extremely low BW neonates to tailor individual therapy during the first weeks of life is a special challenge. A classical dogma ignores elevated serum creatinine values because of maternal creatinine transfer to the neonate during pregnancy. However, when the neonate has impaired renal function, part of this elevation is caused by this impairment. Van Donge et al^2^ developed a mathematical model to characterize serum creatinine concentration and creatinine clearance as a function of GA, mode of delivery, and nephrotoxic treatment, such as ibuprofen. This model allows the derivation of GA-adjusted reference ranges for extremely low BW neonates for normative serum creatinine concentrations to optimize therapy. In addition, this model can quantify drug impact on kidney function after beginning well-known nephrotoxic therapies, such as amikacin and vancomycin.^3^

In addition to the maturation of liver and kidney function, body composition also changes with age. For example, the body of a preterm neonate consists of 80% total body water, whereas a term neonate has 70%, decreasing to 60% by the toddler age. This should have consequences for the volume of distribution for highly water-soluble drugs, as is the case for most antibacterial drugs.

Other factors that strongly influence drug PKs in neonates are extracorporeal life support options, such as extracorporeal membrane oxygenation (ECMO) for pulmonary failure and extracorporeal renal support [continuous renal replacement therapy (CRRT)] for kidney failure. ECMO can be life saving in neonates with cardiac and/or respiratory failure. CRRT is the treatment of choice when the kidneys fail and dialysis is needed. ECMO and CRRT can affect the already altered PKs based on 2 mechanisms: (1) a rapid increase in the volume of distribution because of the volume of the extracorporeal circuit and the hemodilution that occurs and (2) sequestration of the drug in different parts of the circuit. By these 2 mechanisms, a sudden drop in the concentration of the drug can occur, especially when the drug has a small volume of distribution. In addition, changes in drug clearance can occur because of extracorporeal removal (which is the purpose of CRRT) or the binding of the drug to the circuit. In the latter case, a saturation of the binding sites leads to a reduction in virtual extracorporeal clearance over time.

Therefore, in neonates, the “a priori” predictability of treatment outcome is far less than in the adult population. In these cases, if a relationship exists between the concentration of a drug in a bodily fluid, such as blood, serum, plasma, or saliva, and its clinical effect, the measured concentration can be used as a proxy for the effect and to optimize (personalize) the dose.

### Criteria for TDM

Several requirements make the measurement of drug concentration in bodily fluid useful for the optimization of drug therapy.

1. There must be a relationship between the concentration of the drug in a bodily fluid (blood, serum, plasma, and saliva) and its clinical effect or adverse effects.
2. There is no straightforward relationship between the drug dose and its clinical effect or adverse effect.
3. There is a narrow therapeutic range, meaning that the margin between efficacy and toxicity is small.
4. The interpatient variability is larger than the therapeutic range.
5. The clinical effect is difficult to assess (eg, a drug against cancer or a drug against depression may take weeks to months to show an effect).
6. The results need to be properly interpreted, preferably using state-of-the-art software tools, such as Bayesian optimization and population PK models, in concert with pharmacodynamically determined target values and optimal blood sampling. However, there are situations where TDM is not possible.

7. There is no assay for the drug available.
8. The dose cannot easily be adjusted.

Currently, point 7 is less of an issue. In the past, analytical possibilities relied on the availability of commercial assays, such as ligand-binding assays. In special cases, hospitals also use high-performance liquid chromatographic or gas-chromatographic methods to develop their in-house assays, but this is usually only possible in academic and top-clinical hospitals. These classical chromatographic methods lacked sensitivity for the low concentrations of many drugs, and time-consuming preanalytical techniques, such as solid-phase or liquid–liquid extraction and the concentration of the resulting organic layer, were necessary before chromatographic separation and quantification could take place. Consequently, larger sample volumes (usually 0.5–1 mL serum or plasma) are usually required to reach the necessary lower limit of quantitation. This used to be a serious drawback for the widespread introduction of TDM in neonatal care. Currently, liquid chromatography with tandem mass spectrometry (LC-MSMS) has become an affordable technology that is well-established in many hospitals, and it can be used to develop assays for drugs if no commercially available assay exists.^4^ Major points in favor of LC-MSMS are the small sample volumes necessary (usually only 10 µL of serum or plasma) and that simple, fast sample clean-ups, such as simple protein precipitation with a solution containing an internal standard before analysis, can be performed. In addition, the wide availability of stable isotopes of the analytes of interest makes chromatographic separation and sample pretreatment less important because these stable isotopes correct for matrix effects.

For primarily parenterally administered drugs, such as antimicrobials, point 8 is even less relevant.

### Antimicrobial Drugs

Antimicrobial drugs are among the most frequently used drugs in neonates. On average, 36.7% of the hospitalized children receive antimicrobial drugs.^5^ This figure ranges from 12.3% in a general neonatal ward to 61.3% in a pediatric intensive care unit.^5^ Prescribing antimicrobial drugs in neonates is more complex than in adults because of the aforementioned aspects of maturation and extracorporeal life support options, making it a challenge. In addition, most antibiotics have not been well investigated in pediatric patients, especially neonates. Because of ethical considerations, new drugs are usually investigated in adults, and after licensing, neonatal dosing regimens are derived from studies in adults; therefore, no formal SmPC dose advice exists, which means that their use is usually off label. Although techniques, such as allometric scaling and recent models that capture developmental changes in clearance, are used to best predict age-appropriate dosing schemes in neonates,^6,7^ dosing remains an estimation. For some drugs, real-life neonatal PK studies have been published, but these data are usually collected in left-over material during regular off-label use and not in formal PK studies.

Serious infections, such as sepsis, are associated with high morbidity and mortality. In 2002, critical care and infectious disease specialists issued a plan to develop guidelines for treating severe sepsis and septic shock.^8^ Every hour of delay in starting the appropriate treatment increases mortality by 10%, and one of the cornerstones of this “surviving sepsis campaign” was early treatment with an appropriate dose of the right antibacterial drug.^9^

As explained above, newborn neonates have a higher extracellular volume than children and adults.^10^ Most antibiotics are hydrophilic; thus, they are highly water soluble and easily distributed into extracellular compartments, leading to a higher volume of distribution when expressed in liters per kg BW in neonates than in children and adults. This implies that to obtain adequate antimicrobial drug levels quickly, a higher loading dose per kg BW must be administered. Immature clearance results in a lower or less frequent maintenance dose. For some drugs, special neonatal nomograms have been developed based on the dosing guidelines.^11,12^ However, nomograms are still simplifications, and the remaining high intraindividual variability resulting in poor target attainment makes a strong case for TDM.^13^

The goal of this opinion article is to describe the current state of the art of TDM using current analytical techniques and software support to perform optimal model-informed precision dosing of antimicrobial drugs in this special group of patients.

## METHODS

### Literature Search

To obtain information from published original evidence for TDM practices of antimicrobial drugs and outcomes in neonates, PubMed was searched using the following terms: ([“Therapeutic” AND “Drug” AND “Monitoring”] OR “TDM”] AND [“neonate” OR “neonates” AND “Clinical Trial” OR “Clinical Study”]). Then, the retrieved articles were manually selected for antimicrobial drugs.

In addition, the literature was searched for background information on the retrieved classes of drugs. This background information comprised PK/PD background and relevant population PK studies.

## RESULTS

### Literature Search

A total of 447 articles were retrieved. After reviewing the titles and abstracts, 19 articles that discussed antimicrobial drugs were selected. Of these 19 articles, 7 discussed aminoglycosides (gentamicin or netilmicin), 5 articles discussed beta-lactam antibiotics (amoxicillin, flucloxacillin, piperacillin, meropenem, and ceftazidime), 3 discussed glycopeptide drugs (vancomycin and teicoplanin), 2 discussed fluoroquinolones (ciprofloxacin), 1 article discussed MRSA drugs in general, and 1 discussed antimycotic drugs (amphotericin B and caspofungin). After reading the articles, only 2 articles used TDM to adjust doses and evaluated the impact of TDM on future drug concentrations.^14,15^

## DISCUSSION, GAP ANALYSIS, AND OUTLOOK

### Tools for TDM

**FIGURE 1.**
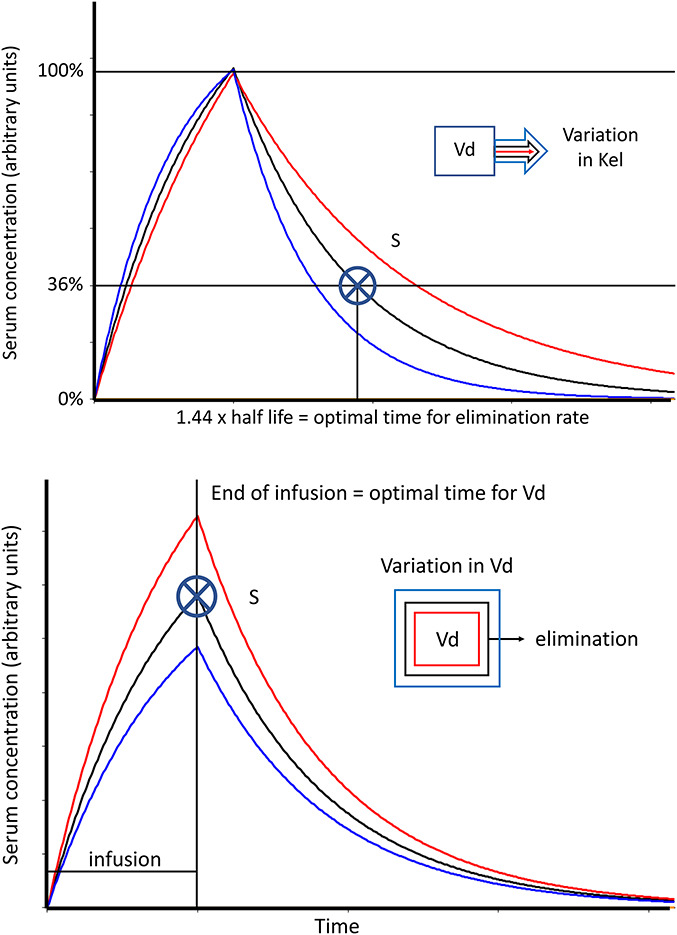
Upper panel: Change in the elimination rate (k_el_) causes the greatest change in the concentration data point S when the latter is at its highest value (true peak concentration). This is the optimal time to calculate V_d_ for a one-compartment model with intermittent intravenous administration. Lower panel: A change in the volume of distribution (V_d_) causes the greatest change in concentration data point S, which is 1.44 half-lives after the end of intravenous administration. This is the optimal time to calculate the k_el_ for a 1-compartment model with intermittent intravenous administration. Adapted with permission from Jelliffe et al.^26^ Clin Pharmacokinet. 1991;21(6):461–478. Adaptations are themselves works protected by copyright. So in order to publish this adaptation, authorization must be obtained both from the owner of the copyright in the original work and from the owner of copyright in the translation or adaptation.

TDM measures drug concentration during the steady state and adjusts the dose accordingly by applying linear PKs. In stable patients, steady-state concentrations will be reached after 4 half-lives. This approach can be applied where applicable. However, in neonates, with immature clearance resulting in longer half-lives that are difficult to predict, doctors must wait longer than in adults before a steady state is reached. In addition, neonates are seldom stable; thus, PKs may change from day to day. The Bayesian population PK modeling software is a valuable tool for TDM in neonates. Software packages for Bayesian TDM first appeared 30 years ago. However, they are operated through the command line and are generally too complicated for widespread use. Publications largely reflect local expertise, and there is currently substantial geographical variability in the use of Bayesian TDM software. However, software technology has greatly improved and became more user-friendly, making Bayesian TDM increasingly feasible for widespread implementation. In 2013, the first overview of available software was published, and new tools have been developed since that review.^16–18^ Examples of widely used software for TDM with some applications used in the pediatric population are MWPharm (eg, flucloxacillin^19^), BestDose (eg, teicoplanin^20^), InsightRx (vancomycin^21^), NextDose (busulfan^22^), and Monolix (eg, vancomycin^23^). The reader is referred to the available literature because this field is developing quickly, and this list will soon be outdated. The core of the available software is a population PK model and a calculation tool, usually a Bayesian estimator. The population PK model is based on the PK parameter values of many individuals with the same characteristics. This will result in mean parameter values and their associated standard deviations for the entire population from which these parameters are derived. In addition, confounders that can be used to further individualize these PK variables can be identified. This information can be obtained from the literature or from doctors' patients who have already been followed by TDM. The Bayesian approach allows adjustment of the dose in the early phase when the steady state has not yet been reached. Early adaptation of the dose allows the achievement of therapeutic goals earlier with higher precision and a better chance for therapeutic success. For example, Van Lent-Evers et al and Bartal et al demonstrated the success of this strategy in adults.^24,25^ An essential aspect for an accurate estimation of actual individual PK parameters and prediction of the correct dose is the optimization of the sampling process. Figure 1 shows that a blood sample drawn immediately after the intravenous administration of a drug provides much information on the volume of distribution (V_d_). However, no information on the elimination rate (k_el_) and a sample drawn 1.44 times the half-life after the end of administration (given as intravenous dose) provides the most information on the k_el_ of the same drug.^26^ When an intravenous drug treatment is started, the time for the maximum drug concentration is easily established, but for the time point that provides the most information on elimination, an estimation based on the average half-life in this population still needs to be made.

To avoid unnecessary blood sampling of neonates, sparse sampling is recommended. Formally, for every PK parameter that is part of a PK model, a separate blood sample needs to be drawn. However, Bayesian software allows prior knowledge regarding the PK parameters in other but similar patients, thereby reducing the number of samples needed to calculate reliable dose adjustments. Furthermore, the principle of D-optimality has been described by Drusano et al and Sallas,^27,28^ and software exists that can calculate optimal sampling times for a given PK model and a reasonable number of samples, such as the design module of the ADAPT II package of the programs of D'Argenio and Schumitzky.^29^ Using optimally drawn samples in combination with Bayesian PK software allows for the attainment of therapeutic targets in a timely manner with a minimal burden on the patient.

### Aminoglycosides

The classic example of TDM in neonates is that of aminoglycosides. Aminoglycosides, such as gentamicin, netilmicin, tobramycin, and amikacin, are among the most widely used antimicrobial drugs in neonates.^5^

The emergence of multiresistant bacteria and the impression that the decline in susceptibility to aminoglycoside antibiotics is less steep than expected has renewed interest in these highly effective and potentially toxic antibiotics. The basic chemical structure required for high potency and a broad spectrum of the antimicrobial activity of aminoglycosides is that of one or several aminated sugars joined by glycosidic linkages to a dibasic cyclitol (2-deoxystreptamine, in most clinically used aminoglycosides).^30^ Aminoglycosides act primarily by impairing bacterial protein synthesis by binding to prokaryotic ribosomes.^30^ Passage of these highly polar molecules across the outer membrane of Gram-negative bacteria is a self-promoted uptake process involving drug-induced disruption of the lipopolysaccharide outer membrane. After penetration through the inner membrane, they bind to the 30S subunit of ribosomes in the cytosol.^30^ This leads to proofreading perturbation of nascent proteins and impaired quality control of the bacterial protein production process with more aberrant proteins inserted into the cell membrane. These actions lead to instability of the outer cell membranes, increased penetration of aminoglycosides, and ultimately cell death.^30^ Through the disruption of cell membranes, aminoglycosides also potentiate the efficacy of beta-lactam antibiotics that also affect cell membrane structure. Although all clinically used aminoglycosides are inhibitors of prokaryotic protein synthesis at commonly accepted therapeutic concentrations, at higher concentrations, they may also affect protein synthesis of mammalian cells, leading to clinically relevant toxicity, including nephrotoxicity, ototoxicity, and vestibulotoxicity.^31^ However, in mammalian cells, it has been demonstrated that aminoglycoside uptake is saturable,^32^ thereby allowing comparatively high concentrations with relatively low toxicity.

Because of their efficacy, aminoglycosides continue to play a valuable role in treating infections caused by aerobic Gram-negative bacteria. However, for optimal use of these agents, it is necessary to understand the PK/PD indices, which are determinants of their therapeutic efficacy and toxicity, to perform rational dose adaptations.

**FIGURE 2.**
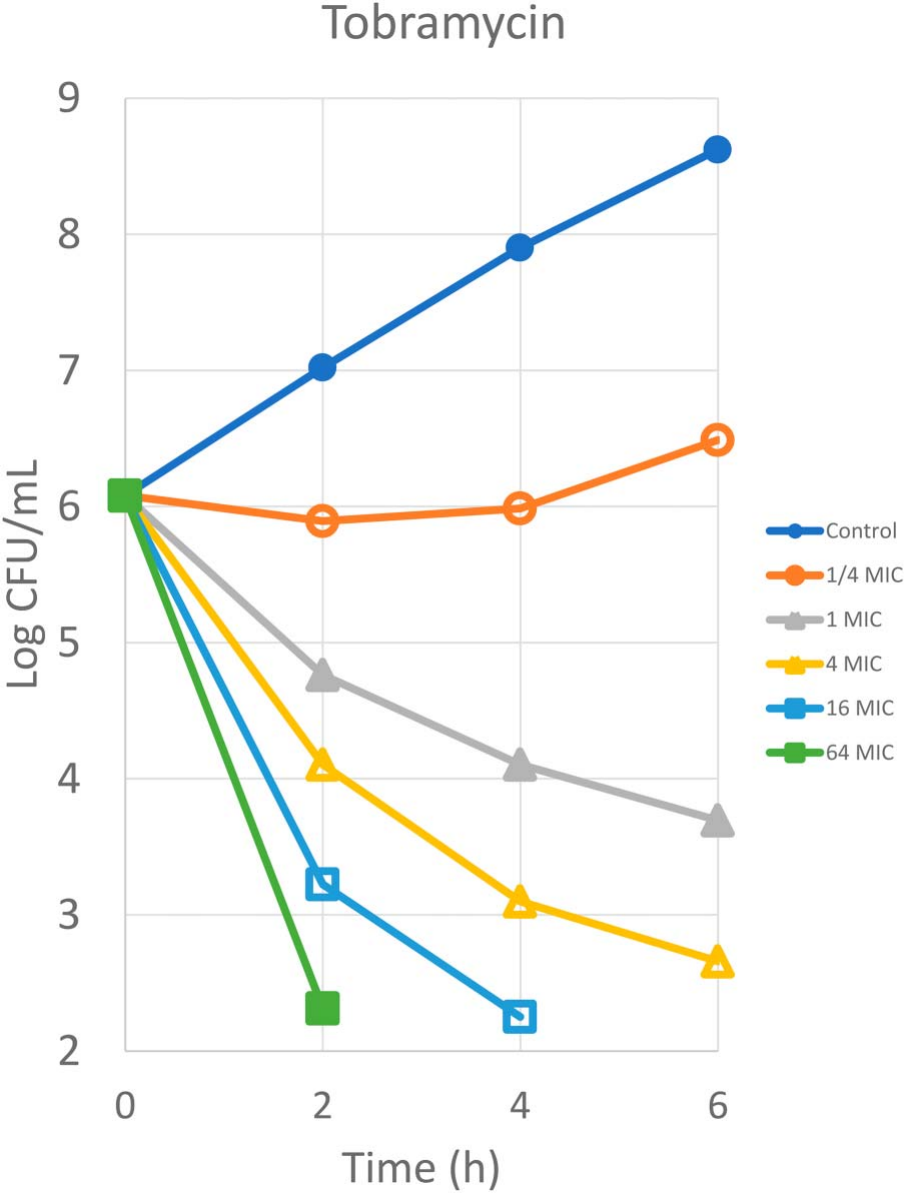
Horizontal axis represents the time of exposure to a certain concentration of the antibiotic. The vertical axis represents the number of colony-forming units. When a microorganism, such as *Pseudomonas aeruginosa*, is exposed to an increasing concentration of tobramycin, its killing action (expressed as a decrease in CFU versus time) increases exponentially. Adapted with permission from Craig et al. Scand J Infect Dis Suppl. 1990;74:63–70. www.tandfonline.com. Adaptations are themselves works protected by copyright. So in order to publish this adaptation, authorization must be obtained both from the owner of the copyright in the original work and from the owner of copyright in the translation or adaptation.

Aminoglycosides are highly effective antimicrobial drugs that display concentration-dependent bactericidal activity.^33,34^ The determinants of efficacy are related to the sensitivity of the infecting microorganism but also to their PK profile. The antibacterial drug concentration (in vitro) where no growth or killing occurs is called the minimal inhibitory concentration (MIC). When aminoglycoside-susceptible microorganisms are exposed to increasing concentrations of an aminoglycoside for fold-MIC, their number expressed as colony-forming units per unit of mass or volume decreases (Fig. 2). Another mechanism of relevance for the dosing regimen is related to the interplay between aminoglycosides and microorganisms. This is a postantibiotic or post-MIC effect. Aminoglycosides exhibit prolonged bacterial killing after clinical concentrations have dropped below the MIC value for the microorganism.^35^

In addition to in vitro and animal studies, clinical studies have shown that higher peak blood concentrations of aminoglycosides are associated with increased survival and better therapeutic responses in patients with Gram-negative infections.^36^ Given these associations, a relationship between the clinical response to aminoglycoside therapy and the ratio of the maximum concentration in the blood of the patient and the MIC for the pathogen has been demonstrated,^34^ and a ratio of at least 8 needs to be achieved. It has also been demonstrated that the (Cmax/MIC) ratio is related to clinical efficacy; however, the area under the curve (AUC) divided by the MIC (AUC/MIC) ratio can be used.^37^ These relationships have led to the concept of administering higher doses for longer dosing intervals. This “once-daily-dosing” concept was first validated and is currently widely accepted in adults^38^ but has also been implemented in children and neonates.^39–42^

In neonates, treatment with an aminoglycoside is usually started with a standard dose based on BW and is “a priori” adjusted for estimated renal function. Typical values for the volume of distribution of the aminoglycosides are 0.41–0.53 L/kg.^13,43^

The target drug concentrations are based on efficacy and toxicity. Based on the principles described above, target concentrations for aminoglycosides must be defined before TDM can be applied. It is clear that no general therapeutic range exists. Every patient has their optimal target concentration, based on the susceptibility of the microorganism, coadministered antibiotics, immune status of the patient, and coadministration of other nephrotoxic or ototoxic drugs. However, when therapy is initiated, these variables may not be known, and the first dose is usually based on population values for the volume of distribution and expected susceptibility of the targeted microorganism. As a result, aminoglycoside target peak concentrations may differ among countries but widely adapted peak concentrations for gentamicin and tobramycin in neonatology range 8–12 mg/L with a predose trough concentration of <1 mg/L.^13^ For amikacin, because of its lower intrinsic antibacterial and toxic effects, target peak levels are >30 mg/L with predose trough levels of 2–5 mg/L.^41^ Together with an average volume of distribution of 0.4–0.5 L/kg, over time this has resulted in initial doses of 5 mg/kg bodyweight for gentamicin,^13,44^ 4 mg/kg for tobramycin,^13^ and 15–20 mg/kg for amikacin.^41^

**FIGURE 3.**
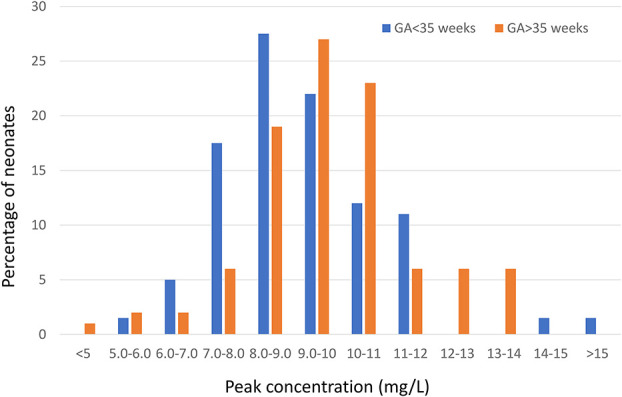
Percentage of neonates within different ranges of peak steady-state concentrations (C_peak_) of gentamicin in the subpopulations GA <35 weeks (N = 64) and GA ≥35 weeks (N = 51). Modified with permission from Sum et al.^44^ Eur J Hosp Pharm. 2007;13(4):98–104. Adaptations are themselves works protected by copyright. So in order to publish this adaptation, authorization must be obtained both from the owner of the copyright in the original work and from the owner of copyright in the translation or adaptation.

However, when a gentamicin dose of 5 mg/kg was administered to a population of neonates, a wide variety of peak concentrations was obtained (Fig. 3).^44^ Further, from this study, it was clear that the stage of maturation (GA) of the neonate played a role. The outcomes of TDM studies largely depend on defined target concentrations. If a wide range of peak concentrations is accepted, the need for TDM is low because most of the concentrations will be in the defined range. However, if narrow target concentrations are defined, there needs to be a substantial effort (TDM) to optimize the therapy. For example, in Figure 3, if the desired peak concentration should be 8–12 mg/L, approximately 80% of the measured peak concentrations fulfill this criterion, and if the required peak concentration is supposed to be > 5 mg/L, 99% of the measured peak concentrations fulfill this criterion. Therefore, using the PK/PD principles described above and considering the risk of toxicity, narrow peak concentrations are currently advocated. This high interindividual variability underlines the need for early intervention after initiating treatment.

Aminoglycosides are eliminated unchanged up to 90% by the kidneys, and only a small portion is metabolized through the liver. Aminoglycosides are filtered by the glomerulus, and there is no evidence of tubular reabsorption or active secretion. Therefore, clearance parallels renal function expressed as the (estimated) glomerular filtration rate (e), except in patients with terminal kidney disease, where the liver plays the most important role in clearance.

Assessment of renal function using plasma creatinine concentration within the first days of life is difficult in clinical practice, as discussed above. Plasma creatinine concentration during this period in the neonate partly reflects the maternal creatinine concentration and is widely neglected. Developmental changes in renal function are reflected in the half-life of any renally cleared drug. Gentamicin, for example, has a half-life of 12–14 hours at a GA of <25 weeks, compared with 6–7 hours at a GA of >32 weeks, whereas clearance increases to 0.41–1.05 mL/kg/min^45^ After birth, irrespective of GA, clearance rapidly increases and half-life decreases.^45^ Based on these prenatal and postnatal maturation–dependent findings regarding the volume of distribution and renal drug clearance, dosing schemes have been developed for drugs with known target peaks and trough concentrations; see Table 1 for an example using gentamicin.^13^

**TABLE 1.** Overview of National Dutch Dose Recommendations for Gentamicin in Preterm and in Term Neonates Depending on GA and PNA

Patient Characteristics	Dose (mg/kg)	Dose Interval (h)
Preterm <32 weeks GA, <7 d PNA	5	48
Preterm 32–37 weeks GA, <7 d PNA	5	36
Preterm, >7 d PNA	4	24
Term, <7 d PNA	4	24
Term, >7 d PNA	4	24
1 month–18 years	7	24

The high interindividual and intraindividual variability stresses the need to optimize the dose immediately after the first dose. This requires a good logistic process for drawing a blood sample, measuring the concentration, and calculating the optimal dose. Taking the first sample immediately after the first dose and the second sample not as a predose trough concentration but 10–12 hours after the first dose facilitates the correct dose and dose interval calculation before the second dose is administered. For optimal dose prediction, Bayesian PK software equipped with the appropriate PK models is required. Successful predictive performance of this early sampling approach has been demonstrated in neonates by Isemann et al.^14^ Currently, the TDM of aminoglycosides in neonates is the standard of practice. A very limited number of studies have been published that have shown beneficial results in neonates; however, it is unethical to perform randomized controlled studies because the PK/PD background and existing evidence in adults are overwhelming.

### Vancomycin

Vancomycin is a glycopeptide antibacterial drug that binds to the cell wall precursor d-alanyl-d-alanine, which is crucial for peptidoglycan crosslinking. Disruption leads to bacterial killing in most Gram-positive species. The most recent guideline of the Infectious Diseases Society of America, issued in 2020, advises to guide vancomycin dosing based on the AUC with a target 24 hours AUC of 400–600 mg·h/L for pediatric patients.^46^ Because the in vitro vancomycin PK/PD index is an AUC/MIC ratio of 400, this target can only be used for microorganisms with a MIC of up to 1 mg/L. However, a recent Chinese study demonstrated that a lower 24 hours AUC target of 240–480 mg·h/L is likely more effective for neonates than adults.^47^ In contrast to aminoglycosides, trough concentrations need to be > 10–15 mg/L to prevent undertreatment because of the absence of a post-MIC effect.

Because vancomycin PKs are determined by the volume of distribution and renal clearance,^48^ neonatal dose recommendations are (similar to aminoglycosides) based on GA and postnatal age, as shown in Table 2.^13^

**TABLE 2.** Overview of National Dutch Dose Recommendations for Vancomycin in Preterm and Term Neonates

Characteristic	Dose (mg/kg)	Dose Interval (h)
Preterm, <7 d PNA	10	12
Preterm, >7 d PNA	10	12
Term, <7 d PNA	8	6
Term, >7 d PNA	12	6
1 month–18 years	15	6

In clinical practice, most clinicians use the predose trough concentration of vancomycin as a surrogate for the 24 hours AUC. Although a simple approach, trough concentration poorly predicts the AUC.^49,50^ However, early high trough concentrations (>20 mg/L) and day one exposure are predictive of clinical outcomes,^50^ which necessitates early (day 1) sampling together with the use of Bayesian software–supported model-informed precision dosing to individualize therapy.^53,54^

In adults, continuous infusion of vancomycin is becoming the standard of practice, especially in ICU units. In the pediatric population, continuous infusion of vancomycin has been studied.^15^ The advantages of continuous infusion are better target attainment and less difficulty in drug monitoring with easier interpretation of drug levels. In addition, AUC targets are reached with fewer dose adjustments and with lower daily doses.^15^

In this study, there was no difference in toxicity between the groups; however, continuous infusion tended to be less toxic in adults.^51^ Currently, the TDM of vancomycin in neonates is the standard of practice. Although limited studies have been published that have shown a benefit of TDM in neonates, it is unethical to perform randomized controlled studies because the PK/PD background and existing evidence in adults in favor of TDM are overwhelming. In addition, Bayesian software with appropriate PK models should be used to individualize and optimize therapy.^52^ As is the case in adults, vancomycin treatment is slowly shifting toward continuous infusion, which facilitates interpretation of drug levels and adjustment of the dose according to the measured concentration and reduces the dose needed to attain the AUC target, which could reduce toxicity.

### Beta-Lactam Antibiotics

Beta-lactam antibiotics act through the direct disruption of the cellular wall of the pathogen. Peptidoglycan is a heteropolymer and an essential component that provides essential mechanical stability to the bacterial cell wall. During bacterial growth and division, peptidoglycan is produced in several stages, and the final stage is the crosslinking of single peptide chains. Crosslinking is accomplished by a transpeptidase enzyme outside of the cell membrane. Because of their structural similarity, beta-lactam antibiotics can inhibit transpeptidase, thereby halting crosslinking and disrupting bacterial cell wall structure and stability. This leads to the lysis of the dividing bacterium. Beta-lactam antibiotics, often used in neonates, are beta-lactamase–sensitive and resistant penicillins, first to fourth generation cephalosporins, and carbapenems.^5^ Fifty to 60% of the prescribed antimicrobial drugs in neonates are beta-lactams.^5^ Beta-lactam antibiotics are mainly eliminated through glomerular filtration and active secretion (eg, flucloxacillin, piperacillin, cephradine, and cefaclor), although some are metabolized in the liver (eg, flucloxacillin). SmPC dosing schemes aim to maintain the plasma concentration of the free (not plasma protein bound) drug for at least 40%–50% of the time above the MIC of the suspected microorganism (fTime > MIC). However, adult studies have demonstrated that in cases of severe infection, the outcome is better if fTime > MIC for 100% of the dosing interval, thereby advocating for either very high intermittent dosing, prolonged infusion, or better administration of beta-lactam antibiotics in the form of a continuous infusion.^53,54^ Because of the high safety level of beta-lactam antibiotics, TDM is rarely performed in clinical practice. However, the question is whether this is justified. Severely ill patients often display different PKs with a much higher volume of distribution and augmented renal clearance (>130 mL/min/1.73 m^2^), leading to the risk of undergoing treatment,^55^ and there is evidence that pediatric dosing strategies for beta-lactam antibiotics are not better.^19^ The added value of TDM is currently under investigation in critically ill adults.^56^ However, some beta-lactam antibiotics can cause neurotoxicity when serum concentrations are too high,^57,58^ limiting irrationally high doses. A common barrier to TDM of beta-lactam antibiotics is because LC-MSMS methods exist for the rapid analysis of these drugs.^4^ Although well documented in adults, PK and PD of these antibiotics are poorly explored in critically ill neonates; the sparsity of studies suggests that current dosing is frequently inadequate.^59–61^ Therefore, there is an urgent need to characterize a population PK of commonly used beta-lactams in neonates associated with target attainment to develop evidence-based dosing schemes and TDM practices. Recently, population PK models for the penicillins, amoxicillin, piperacillin, azlocillin, and the cephalosporin cefathiamidine have been developed and used to calculate target attainment with present SmPC and local dosing schemes.^59–63^ Meropenem is the most widely used carbapenem in neonates.^5^ PKs have been studied in neonates and young children.^64–67^ Compared with adults, total body clearance of meropenem in neonates was comparable; however, the volume of distribution was considerably greater in neonates (38.6 L/70 kg BW versus 22.4 L/70 kg BW).^64,68^ In agreement with aminoglycosides, this implies that higher loading doses per kg BW are needed in neonates for an adequate plasma concentration. These and other population PK models can be used to design rational dosing schemes for neonates and, in combination with optimal sampling schemes for TDM purposes, to further individualize these therapies according to the needs of individual patients.

### Antifungal Drugs

In neonates, the incidence of fungal infections is increasing. Any fungal infection in the neonate can be life-threatening, and a delay in diagnosis often results in significant morbidity or mortality. Currently, amphotericin B is the standard therapy for *Candida* infections. However, amphotericin suffers from significant toxicity, and new antifungal agents, such as echinocandins (eg, caspofungin) or the new generation azole derivatives, have been developed over the past decade. Some clinical experience has been reported regarding the treatment of neonates with *Candida* and *Aspergillus* infections.^69,70^ A prospective study by Mohammed et al investigated the efficacy, safety, and tolerability of caspofungin versus amphotericin B in neonates.^71^ Although retrieved in our search, patients were treated with standard doses of both drugs, and no serum drug concentrations were measured to adjust the therapy.

Based on clinical experience, azoles have also been used in pediatric antifungal infections. Azole antifungal drugs inhibit fungal cytochrome P450 activity, decrease ergosterol synthesis, and inhibit cell membrane formation. The antifungal drug voriconazole is a broad-spectrum triazole agent that is currently the preferred treatment for invasive aspergillosis in adults and children ≥2 years of age. Voriconazole is also effective in the treatment of *Candida* infections. As with many other drugs, voriconazole is currently used off label in children <2 years of age and neonates. Voriconazole exposure has been associated with treatment outcomes in adults, with a suggested cutoff point for voriconazole trough plasma concentrations of 1–5.5 mg/L.^72^ In pediatric patients, an exposure–response relationship was established, in which a voriconazole trough concentration >1 mg/L was associated with improved outcomes.^73^ However, hepatic toxicity can occur at a trough level of >6 mg/L.^74^ Voriconazole is a substrate for CYP2C9, 2C129, and 3A4, displaying developmental and genetic-based variance in PKs. Moreover, voriconazole displays dose-dependent PKs because of the saturation of its metabolism and further inflammation-associated downregulation of metabolic enzymes.^75^ Based on the relationship between voriconazole exposure and efficacy and the resulting high inter-dependent and time-dependent intrapatient variability, the importance of voriconazole TDM has been acknowledged.^76^ Although TDM-based dose adjustments are widely performed in adults to optimize plasma concentrations, it remains unclear whether this method of dose adaptation is used in pediatric patients. Few studies have been performed on pediatric patients, and no studies have been conducted on neonatal patients.^77^ However, in light of the PK difficulties described above and if no other treatment options are available, optimal use of voriconazole guided by TDM is warranted.^77^

Fluconazole is another widely used antifungal drug for neonatal candidiasis. From data extrapolated from adult studies, fluconazole is excreted primarily partly unchanged in the urine and has excellent penetration into the cerebral spinal fluid. For candidiasis treatment, the target area under the concentration curve for 24 hours (AUC24) is ≥ 400 mg·h/L, and with a MIC breakpoint ≤8 mg/L, a ratio of AUC24/MIC >50 mg·h/L is associated with clinical efficacy in adult patients.^78,79^ In contrast to adult data, PK studies in infants have demonstrated that higher doses of 12 mg/kg/d are required to reach these target concentrations.^80^ Although fluconazole is generally considered safe in pediatric patients,^81^ neurotoxicity has been reported at high concentrations.^82^ The combination of a higher dose for comparable exposure and the risk of neurotoxicity makes fluconazole a candidate for TDM.

## CONCLUSION

The goal of TDM is to integrate concentration measurements of a drug as part of clinical decision-making. Aminoglycosides and vancomycin are well-known fields of TDM. However, the upcoming fields for TDM are beta-lactam antibiotics and antifungal drugs, especially voriconazole, because of their poorly predictable PKs. Neonates display wide interindividual variability in PKs and intraindividual variability because of organ maturation immediately after birth and extracorporeal life support factors. This variability hinders good correlations between the dose and concentration of a drug with a serious risk of underdosing, which provides a strong case for goal-oriented, model-informed precision dosing. This is further optimized by measuring one or more drug concentrations that are interpreted using Bayesian PK optimization software. New analytical techniques, such as LC-MSMS, have closed the gap between the need for measurements of serum or plasma drug concentrations and the availability of such assays. Personalized precision dosing cannot be performed without Bayesian software or proper population PK models with confounder identification. Currently, tools available for model development need to be widely adapted to develop validated dosing regimens. In addition, these models can be used with early optimal TDM sampling and Bayesian forecasting to tailor pharmacotherapy to meet the individual needs of neonates.
